# Multidrug-Resistant and Potentially Pathogenic *Escherichia coli* Prevalent in Samples of Different Types of Raw Meat Sold in Informal Markets in Luanda, Angola

**DOI:** 10.3390/foods15010174

**Published:** 2026-01-04

**Authors:** Gomes Cahango, Lélia Chambel, Luisa Brito, Acácio Salamandane

**Affiliations:** 1 LEAF—Linking Landscape, Environment, Agriculture and Food Research Center, Associate Laboratory TERRA, Instituto Superior de Agronomia, Universidade de Lisboa, Tapada da Ajuda, 1349-017 Lisbon, Portugal; gomes.silva.cahango@gmail.com (G.C.); lbrito@isa.ulisboa.pt (L.B.); 2 CNIC—Centro Nacional de Investigação Científica, Ministério do Ensino Superior, Ciência, Tecnologia e Inovação, Avenida Ho Chi Min, Luanda 201, Angola; 3 BioISI—Biosystems and Integrative Sciences Institute, Faculdade de Ciências, Universidade de Lisboa, 1749-016 Lisbon, Portugal; lmchambel@ciencias.ulisboa.pt; 4 Faculdade de Ciências de Saúde, Universidade Lúrio, Campus Universitário de Marrere, Nampula 4250, Mozambique

**Keywords:** informal markets in Luanda, Angola, raw meat, *Escherichia coli*, multidrug resistance (MDR), antimicrobial resistance genes, virulence genes

## Abstract

Raw meat can act as a reservoir and vehicle for antimicrobial-resistant foodborne *Escherichia coli*, particularly when sold under poor hygienic conditions, as is often the case in informal markets in many developing countries, thereby representing a significant public health risk. This study aimed to characterize the antibiotic resistance patterns and virulence of *E. coli* isolated from raw meat sold in informal markets in Luanda, Angola. A total of 99 *E. coli* isolates were recovered from fresh beef, pork and goat meat in five informal markets. DNA macrorestriction analysis by Pulsed-Field Gel Electrophoresis (PFGE) was used to evaluate the genetic diversity of isolates. Multiplex PCR was performed to detect virulent and antibiotic resistance genes. Antibiotic susceptibility was tested using the disk diffusion method. PFGE analysis showed high genotypic diversity. Virulence genes were found in 46% of the isolates, with *astA* (32.3%) being the most frequent. The results also showed high resistance to cefotaxime (67.7%), ampicillin (56.7%) and amoxicillin/clavulanic acid (56.6%). Resistance to imipenem, a last-resort antibiotic, was observed in 30.3% of the isolates. The most prevalent resistance genes were CTX-M group 1 (10.1%) and FOX variants (27.3%). The presence of multidrug-resistant and potentially pathogenic *E. coli* strains in raw meat sold in informal markets in Luanda represents a significant threat to public health. These findings underscore the urgent need to improve surveillance, hygiene practices, and antimicrobial use regulation policies in animal production in Angola.

## 1. Introduction

Meat and meat products are common sources of foodborne pathogenic bacteria, such as *Escherichia coli*, *Staphylococcus aureus*, *Salmonella* and *Listeria monocytogenes* [1]. While many *E. coli* species are harmless commensals, certain pathogenic variants have emerged as a critical public health concern [2]. Among the pathogenic strains, enteropathogenic *E. coli* (EPEC) and Shiga toxin-producing *E. coli* (STEC) are particularly noteworthy due to their prominent role in severe gastrointestinal infections and associated clinical complications. These pathogens are primarily transmitted via the fecal-oral route, occurring through direct contact with infected hosts, both animals or humans, or through consumption of contaminated food and water [3]. Raw meat, particularly beef, pork, and poultry, has long been identified as a common vehicle for the transmission of these enteric pathogenic [4,5,6]. Poor hygiene practices during slaughter and preparation create a persistent transmission pathway for these bacteria, posing a major threat to food safety and human health [7,8,9]. Among livestock-associated pathogens, Shiga toxin–producing *E. coli* (STEC) are particularly concerning, given certain strains, such as EHEC O157:H7, belong to the attaching and effacing (AE-STEC) group and are notorious for causing severe diarrhea, hemolytic uremic syndrome and death [2,10,11]. STEC strains are commonly found in gastrointestinal tracts of asymptomatic ruminants, including cattle, goats, deer, and sheep and frequently contaminate carcasses through fecal shedding during the evisceration process at slaughter [12,13].

Molecular characterization of *E. coli* isolates can provide valuable insights into the genetic basis of pathogenicity and antibiotic resistance. The presence of specific virulence genes, such as those encoding intimin (eae), Shiga toxins (stx1 and stx2), and enterohemolysin (ehxA), is key to identifying pathogenic strains. The emergence of antibiotic resistance among enteric pathogens, including *E. coli*, is a global health threat [11]. Antibiotics are commonly used in the treatment of bacterial infections, but their overuse and misuse in both human and veterinary medicine have led to the development of multidrug-resistant (MDR) strains [12,13]. There is currently no detailed, country-specific quantitative data for Angola publicly available in major global surveillance sources. However, evidence from the wider Sub-Saharan Africa region, where Angola is located, indicates that antibiotics are widely used in animal production for both disease control and growth promotion, often in the absence of strict regulatory oversight [14,15]. Many livestock systems in the region lack robust regulations and effective enforcement governing the sale and use of antibiotics, making non-therapeutic use common, as farmers have easy access to these drugs [15]. Therefore, the investigation of resistance genes, such as blaTEM (resistance to penicillins and cephalosporins) and bla*_CTX-M_* (resistance to cefotaxime), is crucial for assessing the spread of resistance among local strains and determining potential risks to public health. Understanding the molecular features of *E. coli* in food safety contexts is crucial for implementing effective preventive measures, such as better food handling practices, and for informing public health policies aimed at controlling foodborne infections and antimicrobial resistance [16].

In Angola, as in many other developing countries, foodborne diseases represent a major public health concern, with raw meat being one of the most common sources of contamination [17,18]. However, there is limited research on the molecular characterization of *E. coli* strains, particularly regarding pathotypes and antibiotic resistance profiles. This gap is critical since diarrheagenic *E. coli* pathotypes are among the leading causes of morbidity and mortality, especially in children under five years of age, and can contribute to outbreaks of acute gastroenteritis with significant social and economic impacts [19,20,21]. In addition, the increasing prevalence of antimicrobial resistance poses an additional challenge for treatment and control, potentially leading to prolonged illness, higher healthcare costs, and greater risk of complications [22,23,24]. Addressing these issues is essential to strengthening food safety surveillance systems, guiding evidence-based interventions, and mitigating the burden of foodborne infections in Angola and across sub-Saharan Africa.

In this context, this study aims to fill these knowledge gaps through the molecular characterization of *E. coli* isolates recovered from raw meat samples in Luanda, Angola. The results of this research will contribute to the development of targeted strategies to improve food safety and control antibiotic resistance, ultimately contributing to the protection of public health in Angola and other sub-Saharan African countries facing similar challenges.

## 2. Materials and Methods

### 2.1. E. coli Isolates and Identification

A total of 99 *E. coli* isolates recovered between January and April 2024 from three types of fresh meat (beef, pork and goat) in five markets in Luanda were used in this work. The isolates were recovered following the ISO 16649-2 guidelines [25], as detailed in Gomes et al. [26]. Findings from the microbiological analysis of the samples showed that, according to the Angolan government regulation [27], they were unsatisfactory for consumption (*E. coli* > 2.6 log CFU/g) [26]. A well-isolated colony with characteristic *E. coli* traits was chosen from each plate. The presumptive *E. coli* were characterized by biochemical tests, namely Gram staining, catalase and oxidase tests. Confirmatory identification of the *E. coli* isolates was performed via PCR targeting a 401 bp region of the 16S rRNA gene, with primer pair AB035924 (forward: 5′-CCC CCT GGA CGA AGA CTG A-3′; reverse: 5′-ACC GCT GGC AAC AAA GGA T-3′), as previously reported [28]. Subsequently, the amplified PCR products were sequenced to compare in gene bank. The *E. coli* ATCC 25922 was used as a control.

### 2.2. Pulsed Field Gel Electrophoresis (PFGE)

Subtyping of *E. coli* isolates was performed by PFGE of DNA macrorestriction fragments according to PulseNet International [29]. Briefly, the bacterial isolates were first grown onto Tryptone Soy Agar (Biokar Diagnostics, Beauvais, France) and incubated at 37 °C for approximately 14 ± 2 h. Colonies obtained from these plates were then used to prepare bacterial suspensions, which were mixed with SeaKem Gold agarose (Bio-Rad Laboratories, Milan, Italy). The embedded cells were subjected to lysis and washing steps, after which their DNA was digested directly in the plugs using 50 U of the restriction enzyme XbaI (Thermo Fisher Scientific, Fermentas; Waltham, MA, USA) at 37 °C for 3 h. The resulting macrorestriction fragments were separated on 1% (*m*/*v*) SeaKem Gold agarose gels prepared in 0.5 × Tris-Borate-EDTA buffer (Bio-Rad Laboratories, Milan, Italy). Electrophoresis was carried out using a CHEF DRII System (Bio-Rad Laboratories, Milan, Italy) at 14 °C and 6 V/cm with a switch-time ramp of 6.7–35.3 s for 18 h. Gel images were captured with the Gel Doc™ EZ imaging platform (Bio-Rad Laboratories, Milan, Italy). For standardization, TIF files were normalized by matching the XbaI digestion patterns of the reference strain Salmonella Braenderup H9812, which was included in two lanes of every gel.

### 2.3. Antibiotic Susceptibility Profiling

Methods based on disk diffusion on Mueller-Hinton (MH) agar (Biokar Diagnostics, Beauvais, France) were used to evaluate the antibiotic susceptibility profile of the 99 *E. coli* isolates, according to the Clinical Laboratory Standards Institute (CLSI, 2021) [30]. The *E. coli* ATCC 25922 served as quality control. Colonies grown on Trypto-Casein-Soy agar (Biokar Diagnostics, Beauvais, France) at 37 °C for 18 ± 2 h were suspended in ringer solution (Biokar Diagnostics, Beauvais, France) to achieve a turbidity matching the 0.5 McFarland standard (≈10^6^ CFU/mL). These standardized suspensions were then used to inoculate Mueller–Hinton (MH) agar plates, after which four antibiotic disks were placed on each plate and the plates were incubated at 37 °C for 18 ± 2 h. Fifteen antibiotic disks (Liofilchem, Roseto degli Abruzzi, Italy) were tested, grouped by antibiotic class: β-lactams amoxicillin (AMX, 10 µg), amoxicillin/clavulanic acid (AUG, 30:10 µg), ampicillin (AMP, 10 µg), ceftazidime (CAZ, 30 µg), cefotaxime (CTX, 30 µg), cefpirome (CPO, 30 µg), cefoxitin (FOX, 30 µg), imipenem (IPM, 10 µg), aztreonam (ATM, 30 µg); aminoglycosides gentamicin (GEN, 10 µg); tetracycline (TET, 30 µg); chloramphenicol (CHL, 30 µg); sulfonamides trimethoprim/sulfamethoxazole (SXT, 1:19 µg); macrolides azithromycin (AZM, 15 µg); and fluoroquinolones ciprofloxacin (CIP, 5 µg).

### 2.4. Detection of Antibiotic Resistance Genes and Virulence Genes by Multiplex-PCR (MPCR)

To obtain *E. coli* lysates for Multiplex-PCR (MPCR), two or three Colonies obtained from TSA plates after 18 ± 2 h of incubation at 37 °C were suspended in 300 µL of sterilized ultra-pure water and incubated in a boiling water bath for 10 min [31]. After cooling to room temperature, the tubes were centrifuged at 12,000× *g* for 5 min at room temperature (25 °C), and the resulting supernatants (lysates) were stored at −20 °C until further use.

For screening the antibiotic resistance gene, 10 pairs of primer sets were used in four different MPCR reactions, in a total volume of 25 μL each, containing 12.5 µL of Taq DNA Polymerase NZYTaq II2× Colorless Master Mix (MZTech, Lisbon, Portugal). Regarding gene encoding extended-spectrum ß-lactamases (ESBL), a total of five pairs of primers (bla*_TEM_*, bla*_SHV_*, bla*_OXA_*, bla*_CTX-M1_* and bla*_CTX-M9_*) were used (Table 1). For AmpC β-lactamase genes, five pair primers (ACC, FOX, MOX, CIT and DHA) were used (Table 1). All MPCR were performed as previously described in a previous study [32] and subjected to the following amplification program: initial denaturation at 94 °C for 10 min, followed by 30 cycles at 94 °C for 40 s, 60 °C for 40 s, 72 °C for 1 min and a final elongation step at 72 °C for 7 min.

The amplification reactions for screening virulence gene, were carried out in a final volume of 25 µL, which included 12.5 µL of NZYTaq II 2× Colorless Master Mix (Taq DNA Polymerase; MZTech, Lisbon, Portugal). A total of five pairs of primers targeting five genes encoding the respective virulence factors, namely: *stx* (shiga/vero toxin, VT); *lt* (heat-labile enterotoxin, LT); *st* (heat-stable enterotoxin, ST); *astA* (heat-stable enterotoxin 1, EAST1) and *eae* (intimin). The reaction mixture was brought to the final volume with sterile Milli-Q water, and primers (Table 2). PCR amplification consisted of an initial denaturation at 95 °C for 5 min, followed by 35 cycles of 95 °C for 30 s, 50 °C for 40 s, and 72 °C for 1 min, and a final extension step at 72 °C for 10 min.

All MPCR reactions were carried out using a thermocycle GeneAmp^®^ PCR System 9700 thermocycler (Applied Biosystems, Bio-Rad Laboratories, Segrate, Milan, Italy). The amplicons were separated on 1.5% (*m*/*v*) agarose gels prepared in 1 × TAE buffer and run in an EC330 electrophoresis unit (Thermo Fisher Scientific, GA, USA) at 6 V/cm for 120 min. Following electrophoresis, gels were stained with GelRed (Frilabo, Maia, Portugal) and visualized with the Gel Doc™ EZ imaging system (Bio-Rad Laboratories, Segrate, Milan, Italy). The 100 bp DNA Ladder (Invitrogen, CA, USA) served as the molecular size marker for estimating fragment lengths.

### 2.5. Data Analysis and Interpretation

PFGE patterns were compared by BioNumerics^®^ 6.6 (Applied Maths, Kortrijk, Belgium), with a hierarchical numerical process based on the Pearson correlation coefficient (no optimization) and the unweighted pair group method with arithmetic average (UPGMA) as the agglomerative clustering. The reproducibility obtained was determined as the average value for 22 pairs of isolates. The dendrogram was constructed based on 85 isolates with a cut-off at 83.9% (determined reproducibility level).

Antibiotic resistance was evaluated by measuring the inhibition zone diameters (mm) and interpreting them according to CLSI (2021) guidelines [30]. Isolates showing either intermediate or complete resistance were classified as non-susceptible to the corresponding antibiotic. Multidrug resistance was defined as resistance to more than two antimicrobial agents from distinct classes [34].

## 3. Results

### 3.1. Pulsotyping of the Isolates

**Figure 1:**
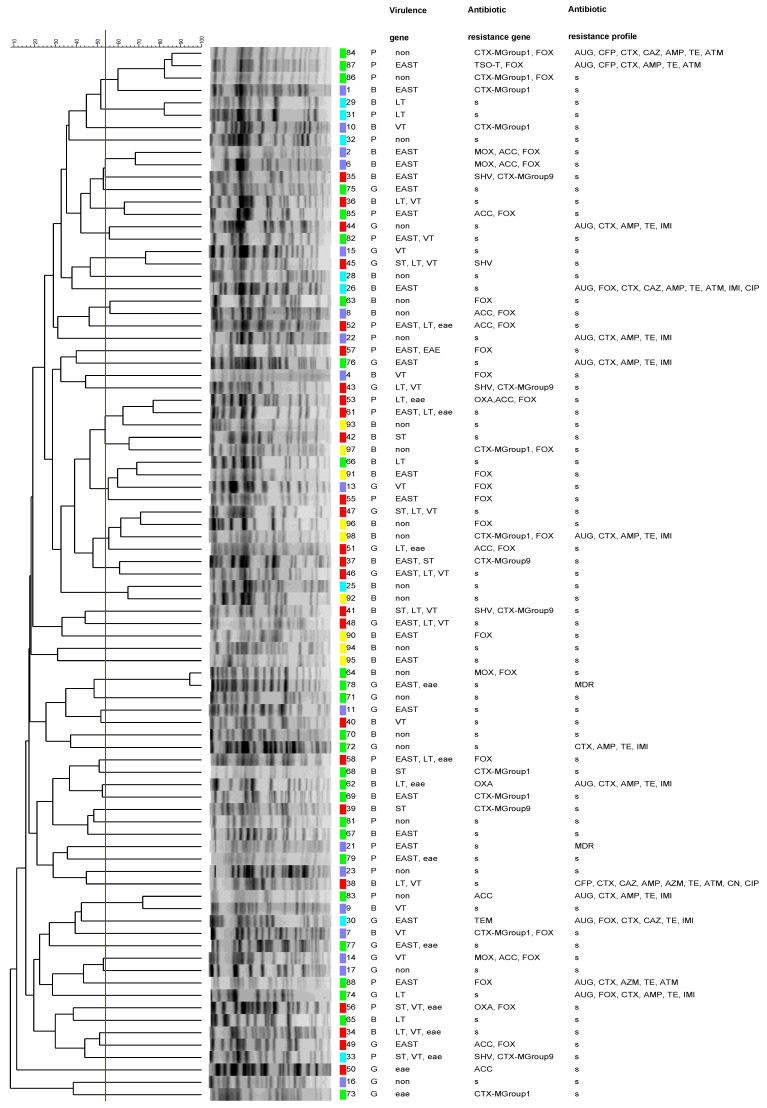
Dendrogram of XbaI-PFGE patterns of 85 *E. coli* isolates. The profiles were compared using BioNumerics^®^ 6.6 (Applied Maths, Kortrijk, Belgium) with a hierarchical numerical process based on Pearson’s correlation coefficient (without optimization) and the unweighted pair group method with arithmetic average (UPGMA) as agglomerative clustering. The red line indicates a reproducibility level of 83.9%, calculated as the mean for 22 isolate pairs. Colored squares distinguish the markets: Km30 (green), Catinton (dark blue), Benfica (red), Kifica (light blue), and Kokolo (yellow). Each isolate is identified by a reference number, along with its market of origin and meat type (P for pork, G for goat, and B for beef). The other columns indicate the presence of virulence genes, antibiotic resistance genes, and the antibiotic resistance profile. non—target genes were not found; s—exhibited susceptibility to all tested antibiotics; MDR—multidrug resistance.

A total of 99 isolates were used in this study. However, in the PFGE, only 85 were presented, since the profiles of the other 14 isolates were not suitable for PFGE analysis. The PFGE analysis of XbaI macrorestriction DNA fragments from 85 *E. coli* isolates generated between 12 and 21 fragments per isolate, with molecular sizes between approximately 20.5 and 1135 kb. Figure 1 illustrates the dendrogram with the 85 isolates that presented a profile suitable for analysis. The descriptive characteristics displayed alongside each isolate were not used in the clustering analysis and therefore did not influence the inferred genetic relationships. The red line indicates the similarity cut-off set at 83.9%, corresponding to the reproducibility threshold of the method. Different colors denote the sampling markets, while letters identify the meat type (P-pork; G-goat and B-beef).

Dendrogram analysis using an 83.9% similarity cut-off revealed a high degree of genetic heterogeneity among the isolates, with most exhibiting distinct genotypic profiles. An exception was observed for isolates 64 (beef) and 78 (goat meat), both collected from the Km 30 market, which clustered together with a similarity of 94.5%. Despite this close genetic relatedness, the isolates differed markedly in their antimicrobial resistance phenotypes: isolate 78 was classified as multidrug-resistant (MDR), whereas isolate 64 remained susceptible to all antibiotics tested. This discrepancy suggests that antimicrobial resistance in these isolates may be driven by mobile genetic elements or recent selective pressures rather than clonal dissemination alone (Figure 1).

In addition, two other isolates from the Km 30 market, isolates 29 (beef) and 31 (pork), clustered with a similarity of 82.2% and exhibited identical genotypic profiles as well as indistinguishable antimicrobial resistance phenotypes, suggesting possible clonal relatedness or a shared contamination source. Conversely, a similar clustering pattern was observed for isolates 84, 86, and 87, all recovered from pork samples collected at the same market, which grouped at a similarity level of 86% but displayed divergent phenotypic resistance characteristics. This finding indicates that, despite close genetic relatedness, phenotypic variation may arise from differential gene expression, acquisition or loss of resistance determinants, or varying selective pressures within the same retail environment (Figure 1).

### 3.2. Antibiotic Resistance Profiles

A total of 99 *Escherichia coli* isolates recovered from fresh meat samples (beef, goat, and pork) collected across five informal markets in Luanda were tested for susceptibility to 12 antimicrobial agents. Overall, the isolates exhibited high levels of resistance to β-lactam antibiotics, with particularly elevated resistance rates observed for CTX (67.7%), AUG (56.6%), AMP (56.7%) and IMI (30.3%). Among the non-β-lactam, tetracycline showed the highest resistance frequency (36.4%). These findings indicate a substantial burden of resistance to commonly used antimicrobials in *E. coli* from informal meat markets, underscoring the potential public health risk associated with the food chain (Table 3).

Regarding the contribution of each market to the overall resistance profile, a high frequency of resistance to AUG was observed in all markets analyzed, especially in the Kikolo (90%), Catinton (83%) and Km 30 (59%) markets. The highest rates of resistance to CTX were recorded in the Kikolo (90%), Km 30 (89%) and Benfica (83%). For AMP, the highest resistance rates were recorded in isolates obtained from the Km 30 (74%), Kikolo (60%) and Catinton (57%) markets (Table 3).

The antimicrobial resistance profile of *E. coli* isolates recovered from beef samples (Table 4) mirrored the overall resistance pattern observed in this study. Notably, resistance to AUG was particularly high, with an overall frequency of 61%, largely driven by isolates from the Catinton (100%) and Kikolo (90%) markets. Similarly, elevated resistance to AMP was observed among beef isolates from Catinton (80%) as well as from Benfica and Km 30 (89% each). High levels of resistance to CTX were also detected, especially in isolates from Kikolo (90%), Kifica (83%), and both Benfica and Km 30 (78% each). In contrast, resistance to non-β-lactam antibiotics was generally low among beef isolates. Nevertheless, tetracycline resistance remained comparatively high in isolates from the Catinton and Kikolo markets (40% each), indicating sustained selective pressure for this antimicrobial in specific retail settings (Table 4).

Among *E. coli* isolates recovered from goat meat, resistance to β-lactam antibiotics was particularly pronounced, with CTX (69%), AUG (58%), AMP (46%) and IMI (31%) showing the highest resistance frequencies (Table 5). Analysis of market-specific contributions revealed that isolates from the Kifica (100%), Catinton (86%), and Km 30 (56%) markets accounted for most of the observed resistance to AUG. In parallel, resistance to CTX was predominantly associated with isolates from the Kifica and Benfica markets (100% each), followed by Km 30 (89%). Notably, goat meat isolates from the Km 30 market exhibited the highest levels of resistance to both AMP (67%) and IMI (56%), suggesting a localized accumulation of resistance determinants in this retail setting. Although resistance to non-β-lactam antibiotics was generally low, TET stood out with a comparatively high resistance frequency of 31% among goat meat isolates, indicating persistent selective pressure for this antimicrobial class (Table 5).

For *E. coli* isolates recovered from pork meat, the highest resistance frequencies were observed for the β-lactam antibiotics CTX (66%), AUG (45%) and AMP (38%), as well as for the non-β-lactam antibiotic TET (45%) (Table 6). Market-level analysis indicated that isolates from the Km 30 (80%) and Catinton (50%) markets contributed most substantially to the observed resistance to AUG. Similarly, elevated resistance to CTX was recorded among pork isolates from the Km 30 (90%), Benfica (70%), and Catinton (50%) markets. Notably, the Km 30 market consistently exhibited the highest resistance levels across multiple antibiotics, including AMP (60%) and IMI (30%), as well as the non-β-lactam TET (60%) and AZM (30%). This pattern suggests that pork sold at this market may represent a particularly important reservoir of multidrug-resistant *E. coli* within the informal retail sector (Table 6).

### 3.3. Presence of Antibiotic Resistance Genes

*CTX-M*–type ESBL genes were the most frequently detected ESBL determinants among the isolates, occurring in 16.2% of cases. Within this group, 10 isolates (10.1%) carried *CTX-M* Group 1 variants, whereas 6 isolates (6.1%) harbored *CTX-M* Group 9 variants. In addition to ESBL genes, AmpC-type β-lactamase determinants were also commonly identified, with FOX variants showing the highest prevalence (27.3%), followed by *ACC* variants (11.1%) (Table 7).

Regarding ESBL genes, the highest prevalence was observed in isolates from the Benfica (11.1%), Km 30 (6.1%), and Catinton (4%) markets. In contrast, β-lactamase ampC genes were most frequent in Catinton (14.1%), Benfica (13.1%), and Km 30 (10.1%) (Table 7). Among these, FOX variants predominated, accounting for 8% of resistance genes in Benfica and 7.1% in both Catinton and Km 30.

### 3.4. Presence of Virulent Genes

Among the virulence genes analyzed, a higher prevalence of *astA* gene was observed (36%) (Table 8). This gene encodes EAST1, a heat-stable enterotoxin typical of enteroaggregative *E. coli* (EAEC). The second most frequent genes were *stx* and *lt*, which encode the verotoxin (VT) and the heat-labile toxin (LT), respectively, each present in 22% of the isolates. The *eae* gene, associated with intimin production, was detected in 17% of the isolates, while the *st* gene, which encodes the heat-stable toxin (ST), was detected in 9% of the isolates, being the least prevalent virulence gene (Table 8).

Combinations of at least two virulence genes were identified in 23 isolates. The most common combinations were *astA* and *eae* (17%). With a prevalence of 13%, the following combinations were found: *astA*, *lt* and *eae*; *st*, *lt* and *stx*; *lt* and *stx*; and *lt* and eae. The combinations *st*, *stx* and *eae;* and *astA* and *lt,* accounted for 8% each. The combinations *astA* and *st*; *astA* and *stx*; *lt*, *stx* and *eae* accounted for 4% each (Figure 1).

## 4. Discussion

DNA macrorestriction fragment analysis by pulsed-field gel electrophoresis (PFGE) was used to assess the genetic diversity of 85 Escherichia coli isolates recovered from fresh meat samples (beef, pork, and goat) collected in five informal markets in Luanda. Using an 83.9% similarity threshold, corresponding to the experimentally determined reproducibility level, PFGE analysis revealed a high degree of genetic diversity among the *E. coli* isolates, with no dominant pulsotype identified. The high genetic diversity of *E. coli* isolated from food matrices has also been previously reported in pigs [35] and in ready-to-eat street food [3]. However, isolates 64 (from beef) and 78 (from goat meat), both obtained from the Km 30 market, exhibited 94.5% genetic similarity. Despite this high homology, their antimicrobial resistance profiles differed: isolate 78 was classified as multidrug-resistant (MDR), whereas isolate 64 showed susceptibility to all antibiotics tested. The results suggest that, although the isolates share a common clonal origin, they likely evolved in distinct environments, acquired different genetic determinants, and accumulated adaptive mutations to withstand specific selective pressures [3,36]. Similar results were reported by Salamandane et al. [3], who found that PFGE clustered isolates with a high level of similarity (>85%), yet they exhibited different virulence genes or distinct antibiotic resistance profiles.

It is noteworthy that isolates 29 (from beef) and 31 (from pork), both obtained from the Kifica market and phenotypically identical, exhibited 82.2% similarity. Although this value falls slightly below the cutoff point of 83.9%, the observed differences are primarily due to band intensity, as both isolates share the same bands and are likely genetically indistinguishable. In contrast, isolates 84 and 87 (both from pork, collected at the Km 30 market) displayed phenotypic differences but demonstrated a similarity of 86.0%. Despite their visual dissimilarity, these isolates share several bands, suggesting a closer genetic relationship than their phenotypic traits might imply. This is an example of how the use of the Pearson correlation coefficient can bring isolates closer together in similarity analysis compared with coefficients based solely on the presence or absence of bands.

Among the virulence genes analyzed, the most prevalent were *astA* (EAST1) (32.3%), *stx* (verotoxin) (19.1%) and *lt* (heat-labile toxin) (17.1%). Co-occurrence of two virulent genes occurred in 23 isolates, with the most frequent combination being *astA* and *lt* (6%). Diarrheagenic *E. coli* pathotypes represent major foodborne pathogens, posing a significant public health burden in low- and middle-income countries [37,38,39]. In Kenya, the prevalence of diarrheagenic *E. coli* has been reported as 23% among children under five years of age and 20% among food animals [40]. Samples from children revealed the presence of Enteroaggregative *E. coli* (12%), Enterotoxigenic *E. coli* (5.3%), Enteropathogenic *E. coli* (3.3%), and mixed infections with Enteroaggregative/Enterotoxigenic and Enteroaggregative/Enteropathogenic *E. coli* (1.3%). Pathogenic strains were also detected in food animals from the children’s homesteads, notably Enteropathogenic *E. coli* in cattle (13%), Enterotoxigenic *E. coli* in goats (4%), and Enterotoxigenic *E. coli* in poultry (3%) [40].

In the present study, *astA*, encoding enteroaggregative heat-stable toxin 1 (EAST1), was the most frequently detected virulence gene (32.3%). This finding agrees with reports from both developing and developed countries, where EAST1-producing *E. coli* has been identified in diverse pathotypes, often as an accessory virulence determinant [41,42]. Similarly, Cho et al. (2020) reported astA as the most prevalent virulence gene (28.3%), followed by escV (18.6%), eaeA (17.7%), and the Shiga toxin genes stx1 and stx2 (3.5% each) [17]. Higher frequencies of astA have also been reported, such as the 53.3% prevalence observed by Sukkua et al. (2017) in *E. coli* isolates recovered from raw meat [43]. In contrast, a study conducted in China on *E. coli* from retail chicken detected only the *eae* gene, and in a single isolate [44].

*E. coli* strains recovered from all meat types and across all markets analyzed in this study exhibited high levels of resistance to several key antibiotics, including amoxicillin–clavulanic acid, cefotaxime, ampicillin, imipenem, and tetracycline. These findings raise public health concerns, as they indicate that antibiotic-resistant *E. coli* is not confined to specific meat categories or market locations, but may be widely disseminated throughout the food chain [45]. The extensive use of antibiotics in animal production, both for therapeutic purposes and as growth promoters, is widely recognized as a major contributing factor to the emergence and spread of bacterial antimicrobial resistance, as it promotes the selection and dissemination of resistant strains, thereby posing a significant threat to public health along the food chain [46,47].

Resistance to β-lactam antibiotics was particularly high for CTX (67.7%), AUG (56.6%) and AMP (56.7%). These findings are consistent with previous studies reporting a high prevalence of β-lactam resistance in *E. coli* strains isolated from food of animal origin [48]. High resistance rates were observed in *E. coli* recovered from retail chicken in China, particularly to TET (84.4%), AMP (71.1%), STX (70.1%) AUG (68.8%) [44]. Antibiotic resistance to AUG and AMP suggests the dissemination of β-lactamase-producing *E. coli*. Even more alarming, resistance to cefotaxime, a third-generation cephalosporin, indicates the possible presence of ESBL–producing strains, which confer resistance to a broad range of β-lactam antibiotics and are frequently associated with therapeutic failure. Notably, resistance to imipenem, a carbapenem typically restricted to hospital use, reached 30.3%, raising concerns regarding the inappropriate use of critically important antimicrobials in livestock production. The detection of imipenem resistance in meat-derived *E. coli* is particularly worrisome, as carbapenems are considered last-resort agents for the treatment of multidrug-resistant infections [49]. This finding may further suggest the presence of carbapenemase-producing Enterobacteriaceae, which are classified by WHO as critical-priority pathogens due to the several limited therapeutic options available [50].

Among the non-β-lactam antimicrobials, TET showed a resistance rate of 36.4%. Its widespread use in animal production is known to favor the selection of resistant strains, underscoring the need for strict regulation of its application [47,51]. Furthermore, the high resistance to TET, one of the most commonly used antibiotics in livestock, reflects the selective pressure exerted by its frequent and sometimes indiscriminate use [52,53]. Collectively, these findings support concerns regarding the role of food-producing animals as reservoirs of antimicrobial-resistant bacteria, which may be transmitted to humans through the food supply chain [16].

High levels of antibiotic resistance in *E. coli* recovered from retail meat have been reported by several authors [49,54,55,56]. In South Africa, *E. coli* recovered from retail meat showed total resistance to erythromycin (100%) and high resistance to several other antibiotics, including cefotaxime (95.58%), ampicillin (88.23%), cefuroxime (88.23%), trimethoprim-sulphamethoxazole (88.23%), tetracycline (60.29%) and imipenem (50%) [49]. Such data highlight the escalating challenge of antimicrobial resistance within the sub-Saharan African region. While data documenting antibiotic resistance in Angolan livestock farming remains scarce, clinical evidence suggests a significant challenge. For instance, *E. coli* strains isolated from human urine at the Luanda Medical Center exhibited total resistance (100%) to cephalexin and cefuroxime, with high resistance rates also recorded for ceftriaxone (92%), gentamicin (92%), amoxicillin-clavulanic acid (80%), and ciprofloxacin (72%) [57]. Kieffer et al. report that 36.3% of *E. coli* recovered from hospitalized children in Luanda were imipenem-resistant [58]. In Congo, clinical *E. coli* showed high resistance to ceftazidime (65%), followed by amoxicillin (57%) and piperacillin–tazobactam (51%) [59].

Regarding meat types, beef showed the highest AUG resistance (100% in Catinton; 90% in Kikolo). Resistance in goat meat reached 60% in Catinton, while pork from Km 30 showed high resistance to both AUG (80%) and CTX (90%). These variations likely reflect different management practices and antimicrobial exposure across species. Given that *E. coli* is a major driver of global morbidity and mortality [60,61], its prevalence in meat at unsatisfactory levels [26] represents a significant threat to public health, especially when associated with multidrug-resistant genotypes.

In the present study, ESBL-associated *bla_CTX-M_* were the most frequently detected (16.2%), with bla*_CTX-M-1_* (10.1%) and bla*_CTX-M-9_* (6.1%) being the predominant groups. These results are consistent with several studies that point to the global dissemination of these genes in isolates of animal origin [48]. In a study conducted on poultry in the Philippines, the most prevalent *bla_CTX-M_* were *bla_CTX-M-1_* (72.46%), followed by *bla_CTX-M-2_* (65.22%) and *bla_CTX-M-9_* group (52.17%) [62]. Among ESBL-positive *E. coli* from Bavarian dairy and beef cattle, bla*_CTX-M_* genes were detected in 93.4% of strains, with bla*_CTX-M group 1_* being the group most frequently found [63]. Several studies have confirmed a higher prevalence of bla*_CTX-M_* genes among commensal *E. coli* recovered from clinical [58] and non-clinical environments in Angola [64,65]. In Uganda, *E. coli* recovered from hematologic cancer patients with bacteremia showed ESBL-encoding genes (bla*_CTX-M_*, bla*_TEM_*, and bla*_SHV_*) in 75% of the samples, and bla*_CTX-M_* was the most common ESBL-encoding gene identified with 91% [66]. Among the AmpC genes, FOX was the most prevalent (27.3%), followed by ACC (11.1%). In Nigeria, AmpC FOX genes were detected in 6.3% of 48 *E. coli* isolates recovered from abattoir samples [67].

More than 56% of the isolates analyzed in this study harbored both virulence and antimicrobial resistance genes, highlighting a significant public health concern. This finding is of significant concern to public health. The co-occurrence of these determinants suggests that raw meat may serve as a reservoir of *E. coli* strains with enhanced pathogenic potential and reduced treatment options. Such strains may give rise to infections that are not only more severe but also more challenging to treat, emphasizing the importance of monitoring and controlling antimicrobial resistance in the food chain. The simultaneous presence of virulence and resistance genes also suggests the potential involvement of mobile genetic elements, such as plasmids and integrons, in facilitating their dissemination throughout the food chain [68]. Supporting this, a study of street food isolates reported that 63% of antimicrobial-resistant strains carried one or more resistance genes alongside virulence determinants [3].

These findings underscore the urgent need to implement public policies aimed at the rational use of antimicrobials in animal production, as well as the strengthening of sanitary inspection systems. Mitigation strategies, such as systematic surveillance of bacterial resistance in food and promotion of sustainable production practices, are essential to contain the dissemination of MDR microorganisms and to mitigate risks to human health.

## 5. Conclusions

This study highlights the presence of *E. coli* exhibiting virulence and multidrug resistance characteristics in raw meat samples sold in informal markets in Luanda, Angola. The detection of clinically relevant resistance genes, such as CTX-M and ampC (especially FOX and ACC variants), along with the prevalence of virulence genes such as *astA*, *stx* and *lt*, signals the potential risk of zoonotic transmission of resistant and pathogenic strains to humans. The high genotypic diversity observed through PFGE indicates that contamination arises from multiple sources, reflecting inadequate hygienic practices throughout the meat supply chain. These findings emphasize the critical need in Angola and other developing countries to implement effective food safety measures, strengthen the rational use of antimicrobials in livestock farming, and develop public health policies focused on monitoring and mitigating antibiotic resistance in the food sector.

## Figures and Tables

**Table 1:** Primers targeting genes encoding antibiotic resistance [33].

Name of Primer	Nucleotide Sequence	Target Gene	Size (bp)	Final Concentration (µM)
MultiTSO-T-For	5′-CATTTCCGTGTCGCCCTTATTC-3′	TEM variants (TEM-1 and TEM-2)	800	0.4
MultiTSO-T-Rev	5′-CGTTCATCCATAGTTGCCTGAC-3′			
MultiTSO-S-For	5′-AGCCGCTTGAGCAAATTAAAC-3′	SHV variants (including SHV-1)	713	0.4
MultiTSO-S-Rev	5′-ATCCCGCAGATAAATCACCAC-3′			
MultiTSO-O-For	5′-GGCACCAGATTCAACTTTCAAG-3′	OXA-variants (OXA-1, OXA-4, OXA-30)	564	0.4
MultiTSO-O-Rev	5′-GACCCCAAGTTTCCTGTAAGTG-3′			
CTX-MGrp1-For	5′-TTAGGAARTGTGCCGCTGYA-3′	Multi CTX-MGroup1 (CTX-M-1, CTX-M-3, CTX-M-15)	688	0.4
CTX-MGrp1-Rev	5′-CGATATCGTTGGTGGTRCCAT-3′			
CTX-MGrp9-For	5′-TCAAGCCTGCCGATCTGGT-3′	CTX-M—Group 9 (CTX-M-9, CTX-M-14)	561	0.4
CTX-MGrp9-Rev	5′-TGATTCTCGCCGCTGAAG-3′			
MultiACC-For	5′-CACCTCCAGCGACTTGTTAC-3′	ACC variants (ACC-1 and ACC-2)	346	0.2
MultiACC-Rev	5′-GTTAGCCAGCATCACGATCC-3′			
MultiFOX-For	5′-CTACAGTGCGGGTGGTTT-3′	FOX variants FOX-1 to FOX-5	162	0.5
MultiFOX-Rev	5′-CTATTTGCGGCCAGGTGA-3′			
MultiMOX-For	5′-GCAACAACGACAATCCATCCT-3′	MOX variants MOX-1, MOX-2, CMY-1, CMY-8 to CMY-11, and CMY-19	895	0.2
MultiMOX-Rev	5′-GGGATAGGCGTAACTCTCCCAA-3′			
MultiCIT-For	5′-CGAAGAGGCAATGACCAGAC-3′	CIT variants LAT-1 to 3, BIL-1, CMY-2 to 7, CMY-12 to -18, and CMY-21–23	538	0.3
MultiCIT-Rev	5′-ACGGACAGGGTTAGGATAGY-3′			
MultiDHA_For	5′-TGATGGCACAGCAGGATATTC-3′	DHA variants	997	0.5
MultiDHA-Rev	5′-GCTTTGACTCTTTCGGTATTCG-3′			

**Table 2:** Primers targeting virulent genes [33].

Primer	Sequence	Target Gene/ (Virulence Factor)	Size (bp)	Final Concentration
LT-For	5′-ATT TAC GGC GTT ACT ATC CTC-3′	*lt* (LT)	280	0.4 µM
LT-Rev	5′-TTT TGG TCT CGG TCA GAT ATG-3′			
ST-For	5′-TCT GTA TTG TCT TTT TCA CC-3′	*st* (ST)	195	0.4 µM
ST-Rev	5′-TTA ATA GCA CCC GGT ACA AGC-3′			
EAE-For	5′-ACC AGA TCG TAA CGG CTG CCT-3′	*eae* (Intimin)	499	0.4 µM
EAE-Rev	5′-AGT TTG GGT TAT AAC GTC TTC ATT G-3′			
ES-For	5′-GAG CGA AAT AAT TTA TAT GT-3′	*stx* (VT)	323	0.4 µM
ES-Rev	5′-CGA AAT CCC CTC TGT ATT TGC C-3′			
EAST 11-For	5′-CCA TCA ACA CAG TAT ATC CGA-3′	*astA* (EAST1)	114	0.4 µM
EAST 11-Rev	5′-GGT CGC GAG TGA CGG CTT TGT-3′			

**Table 3:** Profiles of antibiotic susceptibility of 99 *E. coli* recovered from fresh meat.

Group	Antibiotic	Isolates From Market					
		Catinton	Kifika	Benfica	Km30	Kikolo	Total
β-lactams	AUG (20/10 μg)	19/23 (83%)	3/10 (30%)	9/29 (31%)	16/27 (59%)	9/10 (90%)	56/99 (56.6%)
	FOX (30 μg)	1/23 (4%)	2/10 (20%)	4/29 (14%)	5/27 (19%)	1/10 (10%)	13/99 (13.1%)
	CFP (75 μg)	3/23 (13%)	1/10 (10%)	1/29 (3%)	2/27 (7%)	0/10 (0%)	7/99 (7%)
	CTX (30 μg)	4/23 (17%)	6/10 (60%)	24/29 (83%)	24/27 (89%)	9/10 (90%)	67/99 (67.7%)
	CAZ (30 μg)	1/23 (4%)	4/10 (40%)	6/29 (21%)	2/27 (7%)	0/10 (0%)	13/99 (13.1%)
	AMP (10 μg)	13/23 (57%)	4/10 (40%)	13/29 (45%)	20/27 (74%)	6/10 (60%)	56/99 (56.7%)
	ATM (30 μg)	0/23 (0%)	2/10 (20%)	1/29 (3%)	2/27 (7%)	0/10 (0%)	5/99 (5.1%)
	IMI (10 μg)	3/23 (13%)	5/10 (50%)	5/29 (17%)	14/27 (52%)	3/10 (30%)	30/99 (30.3%)
Non-β-lactams	AZM (15 μg)	1/23 (4%)	0/10 (0%)	5/29 (17%)	3/27 (11%)	0/10 (0%)	9/99 (9.1%)
	TET (30 μg)	10/23 (43%)	4/10 (40%)	6/29 (21%)	12/27 (44%)	4/10 (40%)	36/99 (36.4%)
	CN (10 μg)	1/23 (4%)	0/10 (0%)	1/29 (3%)	0/27 (0%)	0/10 (0%)	2/99 (2%)
	CIP (5 μg)	1/23 (4%)	2/10 (20%)	1/29 (3%)	1/27 (4%)	0/10 (0%)	5/99 (5.1%)

Amoxicillin + Clavulanic acid (AUG); Cefoxitin (FOX); Cefoperazone (CFP); Cefotaxime (CTX); Ceftazidime (CAZ); Ampicillin (AMP); Aztreonam (ATM); Imipenem (IMI); Azitrhomycin (AZM); Tetracycline (TET); Gentamicin (CN) and Ciprofloxacin (CIP).

**Table 4:** Profile of antibiotic susceptibility of 44 *E. coli* isolates from beef.

Group	Antibiotic	Beef/Markets					
		Catinton	Kifika	Benfica	Km 30	Kikolo	Total
β-lactams	AUG (20/10 μg)	10/10 (100%)	2/6 (33%)	3/9 (33%)	3/9 (33%)	9/10 (90%)	27/44 (61%)
	FOX (30 μg)	0/10 (0%)	1/6 (17%)	1/9 (11%)	2/9 (22%)	1/10 (10%)	5/44 (11%)
	CFP (75 μg)	1/10 (10%)	0/6 (0%)	1/9 (11%)	0/9 (0%)	0/10 (0%)	2/44 (5%)
	CTX (30 μg)	1/10 (10%)	5/6 (83%)	7/9 (78%)	7/9 (78%)	9/10 (90%)	29/44 (66%)
	CAZ (30 μg)	1/10 (10%)	3/6 (50%)	4/9 (44%)	0/9 (0%)	0/10 (0%)	8/44 (18%)
	AMP (10 μg)	8/10 (80%)	3/6 (50%)	8/9 (89%)	8/9 (89%)	6/10 (60%)	33/44 (75%)
	ATM (30 μg)	0/10 (0%)	2/6 (33%)	1/9 (11%)	0/9 (11%)	0/10 (0%)	3/44 (7%)
	IMI (10 μg)	1/10 (10%)	3/6 (50%)	2/9 (22%)	6/9 (67%)	3/10 (30%)	15/44 (34%)
Non-β-lactams	AZM (15 μg)	0/10 (0%)	0/6 (0%)	5/9 (55%)	0/9 (0%)	0/10 (0%)	5/44 (11%)
	TET (30 μg)	4/10 (40%)	3/6 (50%)	1/9 (11%)	2/9 (22%)	4/10 (40%)	14/44 (31%)
	CN (10 μg)	0/10 (0%)	0/6 (0%)	1/9 (11%)	0/9 (0%)	0/10 (0%)	1/44 (2%)
	CIP (5 μg)	0/10 (0%)	2/6 (33%)	1/9 (11%)	0/9 (0%)	0/10 (0%)	3/44 (7%)

Amoxicillin + Clavulanic acid (AUG); Cefoxitin (FOX); Cefoperazone (CFP); Cefotaxime (CTX); Ceftazidime (CAZ); Ampicillin (AMP); Aztreonam (ATM); Imipenem (IMI); Azitrhomycin (AZM); Tetracycline (TET); Gentamicin (CN) and Ciprofloxacin (CIP).

**Table 5:** Profile of antibiotic susceptibility of 26 *E. coli* isolates from goat meat.

Group	Antibiotic	Goat/Markets				
		Catinton	Kifika	Benfica	Km 30	Total
β-lactams	AUG (20/10 μg)	6/7 (86%)	1/1 (100%)	3/9 (33%)	5/9 (56%)	15/26 (58%)
	FOX (30 μg)	0/7 (0%)	1/1 (100%)	1/9 (11%)	2/9 (22%)	4/26 (15%)
	CFP (75 μg)	0/7 (0%)	0/1 (0%)	0/9 (0%)	0/9 (0%)	0/26 (0%)
	CTX (30 μg)	0/7 (0%)	1/1 (100%)	9/9 (100%)	8/9 (89%)	18/26 (69%)
	CAZ (30 μg)	0/7 (0%)	1/1 (100%)	1/9 (11%)	1/9 (11%)	3/26 (12%)
	AMP (10 μg)	3/7 (43%)	0/1 (0%)	3/9 (33%)	6/9 (67%)	12/26 (46%)
	ATM (30 μg)	0/7 (0%)	0/1 (0%)	0/9 (0%)	0/9 (0%)	0/26 (0%)
	IMI (10 μg)	0/7 (0%)	1/1 (100%)	2/9 (22%)	5/9 (56%)	8/26 (31%)
Non-β-lactams	AZM (15 μg)	0/7 (0%)	0/1 (0%)	0/9 (0%)	0/9 (0%)	0/26 (0%)
	TET (30 μg)	2/7 (29%)	1/1 (100%)	1/9 (11%)	4/9 (44%)	8/26 (31%)
	CN (10 μg)	0/7 (0%)	0/1 (0%)	0/9 (0%)	0/9 (0%)	0/26 (0%)
	CIP (5 μg)	0/7 (0%)	0/1 (0%)	0/9 (0%)	1/9 (11%)	1/26 (4%)

Amoxicillin + Clavulanic acid (AUG); Cefoxitin (FOX); Cefoperazone (CFP); Cefotaxime (CTX); Ceftazidime (CAZ); Ampicillin (AMP); Aztreonam (ATM); Imipenem (IMI); Azitrhomycin (AZM); Tetracycline (TET); Gentamicin (CN) and Ciprofloxacin (CIP).

**Table 6:** Profile of antibiotic susceptibility profiles of 40 *E. coli* isolates from pork.

Group	Antibiotic	Pork/Markets				
		Catinton	Kifika	Benfica	Km 30	Total
β-lactams	AUG (20/10 μg)	3/6 (50%)	0/3 (0%)	2/10 (20%)	8/10 (80%)	13/29 (45%)
	FOX (30 μg)	1/6 (17%)	0/3 (0%)	1/10 (10%)	1/10 (10%)	3/29 (10%)
	CFP (75 μg)	2/6 (33%)	1/3 (33%)	0/10 (0%)	2/10 (20%)	5/29 (17%)
	CTX (30 μg)	3/6 (50%)	0/3 (0%)	7/10 (70%)	9/10 (90%)	19/29 (66%)
	CAZ (30 μg)	0/6 (0%)	0/3 (0%)	1/10 (10%)	1/10 (10%)	2/29 (7%)
	AMP (10 μg)	2/6 (33%)	1/3 (33%)	2/10 (20%)	6/10 (60%)	11/29 (38%)
	ATM (30 μg)	0/6 (0%)	0/3 (0%)	0/10 (0%)	2/10 (20%)	2/29 (7%)
	IMI (10 μg)	2/6 (33%)	1/3 (33%)	1/10 (10%)	3/10 (30%)	7/29 (24%)
Non-β-lactams	AZM (15 μg)	1/6 (17%)	0/3 (0%)	0/10 (0%)	3/10 (30%)	4/29 (14%)
	TET (30 μg)	4/6 (67%)	0/3 (0%)	3/10 (30%)	6/10 (60%)	13/29 (45%)
	CN (10 μg)	1/6 (17%)	0/3 (0%)	0/10 (0%)	0/10 (0%)	1/29 (3%)
	CIP (5 μg)	1/6 (17%)	0/3 (0%)	0/10 (0%)	0/10 (0%)	1/29 (3%)

Amoxicillin + Clavulanic acid (AUG); Cefoxitin (FOX); Cefoperazone (CFP); Cefotaxime (CTX); Ceftazidime (CAZ); Ampicillin (AMP); Aztreonam (ATM); Imipenem (IMI); Azitrhomycin (AZM); Tetracycline (TET); Gentamicin (CN) and Ciprofloxacin (CIP).

**Table 7:** Prevalence of β-lactam antibiotic resistance genes in non-susceptible *E. coli* isolates.

Subgroup	Antibiotic-Resistant Gene Variants	Number of Isolates (%)					
		Catinton	Kifica	Benfica	Km 30	Kikolo	Total
ESBL	BlaTEM	0	1 (1%)	0	1 (1%)	0	2 (2%)
	BlaOXA	0	0	2 (2%)	1 (1%)	0	3 (3%)
	BlaSHV	0	1 (1%)	4 (4%)	0	0	5 (5.1%)
	BlaMCTX-M1	4 (4.04%)	0	0	4 (4%)	2 (2%)	10 (10.1%)
	BlaMCTX-M G9	0	1 (1%)	5 (5.1%)	0	0	6 (6.1%)
	Total ESBL genes	4 (4%)	3 (3%)	11 (11.1%)	6 (6.1%)	2 (2%)	26 (26.3%)
β-lactam ampC	BlaACC variants	4 (4.04%)	0	5 (5.1%)	2 (2%)		11 (11.1%)
	BlaFOX	7 (7.07%)	0	8 (8.08%)	7 (7.1%)	5 (5.1%)	27 (27.3%)
	BlaMOX	3 (3.03%)	0	0	1 (1%)	0	4 (4%)
	BlaCIT	0	0	0	0	0	
	BlaDHA	0	0	0	0	0	
	Total ampC genes	14 (14.1%)	0	13 (13.1%)	10 (10.1%)	5 (5.1%)	42 (42.4%)
	Total β-lactam encoding genes	18 (18.2%)	3 (3%)	24 (24.2%)	16 (16.2%)	7 (7.1%)	68 (68.7%)

**Table 8:** Prevalence of virulence genes in 99 *E. coli* isolates.

Virulence Gene	Number of Isolates (%)					
	Catinton	Kifika	Benfica	Km 30	Kikolo	Total
*astA*	6/23 (26%)	2/10 (20%)	12/29 (41%)	12/27 (44%)	4/11 (36%)	36/99 (36%)
*st*	0/23 (0%)	1/10 (10%)	7/29 (24%)	1/27 (4%)	0/11 (0%)	9/99 (9%)
*stx*	8/23 (34%)	1/10 (10%)	11/29 (38%)	1/27 (4%)	1/11 (9%)	22/99 (22%)
*lt*	0/23 (0%)	2/10 (20%)	16/29 (55%)	4/27 (15%)	0/11 (0%)	22/99 (22%)
*eae*	0/23 (0%)	1/10 (10%)	11/29 (38%)	5/27 (18%)	0/11 (0%)	17/99 (17%)

## Data Availability

The original contributions presented in the study are included in the article, further inquiries can be directed to the corresponding author.
